# Evaluation of preventive methods used to interrupt the progression of early caries lesions

**DOI:** 10.4317/jced.62588

**Published:** 2025-10-17

**Authors:** Ana Paula Lima da Silva, Taynara Pereira de Oliveira, Ruchele Dias Nogueira, Cesar Penazzo Lepri, Maria Angelica Hueb Menezes de Oliveira, Regina Guenka Palma-Dibb, Vinicius Rangel Geraldo-Martins

**Affiliations:** 1 School of Dentistry - Universidade de Uberaba (Department of Clinical Dentistry), Uberaba (MG), Brazil; 2 School of Dentistry - Universidade de Uberaba (Department of Biopathology), Uberaba (MG), Brazil; 3 Ribeirao Preto Shool of Dentistry – São Paulo University (Department of Restorative Dentistry), Ribeirao Preto (SP), Brazil

## Abstract

**Background:**

The objective was to evaluate the surface hardness of white spot lesions (WSL) treated with different caries prevention methods.

**Material and Methods:**

The Knoop microhardness (µKH) of 50 bovine dental enamel fragments was evaluated. WSL were induced (pH cycling) in those fragments and, after µKH analysis, the specimens received the following treatments (n=10): G1- fluoride gel (NaF 2%), G2- fluoride varnish (NaF 5%), G3-brushing with CPP-ACP based dentifrice, G4- infiltrant resin (Icon-DMG) and G5-Er,Cr:YSGG laser (8.92 J/cm2, 0.5 W). A second cariogenic challenge was done after treatments, and surface microhardness was measured before and after treatments. The Knoop hardness (KHN) values obtained were analyzed by one-way ANOVA followed by Tukey´s test (=5%).

**Results:**

After treatments, the KHN was similar to the baseline in all groups. After the second cariogenic challenge, it was observed that G1, G4 and G5 presented KHN similar to their baseline numbers, and in G2 and G3 the KHN was similar to the post-treatment numbers. In general, the recovery of enamel hardness after treatment was above 86%, whith fluoride gel, varnish and Icon achieving slightly better results than the CPP-ACP and Er,Cr:YSGG laser. The analysis of the acid resistance of WSL showed that in groups 1, 3 and 5 the loss of hardness was lower than that observed in G2 and G4.

**Conclusions:**

It was concluded that the treatments recovered the hardness of the demineralized enamel and that, despite the methods tested did not prevent demineralization of WSL, their effectiveness was greater than 85%.

## Introduction

Dental caries is a dynamic, multifactorial disease, mediated by bacterial biofilm that promotes localized destruction of dental hard tissues and affects a large part of the population (1). Caries can occur throughout life, both in primary and permanent dentition, and can damage the tooth crown and, subsequently, the exposed root surfaces. Its origin depends on the correlation between primary determining factors, represented by dental biofilm, cariogenic microbiota, sugar-based diet and time (2). Initial caries lesions, known as white spot lesions (WSL), are characterized by the presence of opaque areas on enamel, originated through the loss of minerals from the subsurface layer of the hard tissue, without clinically visible cavitation (2). Early diagnosis of enamel demineralization allows the adoption of remineralization therapy, which often halts the progression of the lesion. Currently, fluoride-based compounds are the primary approach for preventing dental caries (4). Their protective effect has been extensively studied in recent years and applied in various products, such as toothpastes, varnishes, gels, and mouthwashes, integrating preventive oral health programs (1 , 2 , 4). However, in cases of incipient carious lesions, fluoride primarily acts on the superficial enamel layer, while the subsurface layer remains mineral-deficient, resulting in limited remineralization (5). Moreover, the effectiveness of fluoride therapy in preventing and controlling caries progression depends on maintaining adequate concentrations in saliva, requiring continuous use of fluoride-containing compounds (6). In light of this, different therapeutic approaches, such as infiltrant resins, lasers, and other technologies, have been investigated as complementary alternatives (6). Casein, a milk-derived protein, has been widely studied for its anticariogenic activity, mainly due to its ability to stabilize calcium and phosphate ions on the tooth surface, creating a supersaturated environment that favors enamel remineralization [7). Amorphous calcium phosphate (ACP) plays a role in maintaining the mineral balance of the tooth surface, as its highly soluble structure facilitates the controlled release of calcium and phosphate ions, promoting a state of supersaturation at the tooth-saliva interface (8). The infiltrant resin is a technique used for minimally invasive treatment, as its low viscosity and high penetration capacity allow it to infiltrate the body of the white spot lesion and fill the porosities, preventing the diffusion of acids that would lead to further demineralization (9 , 10). High-intensity lasers have also been evaluated to increase the acid resistance of tooth enamel. Literature reports have demonstrated that irradiation of dental enamel with high-intensity lasers has promoted a significant reduction in mineral loss (11 , 12). Different hypotheses have been proposed to explain the changes in enamel that result in the preventive effect, and all of them are related to the increase in temperature on the irradiated tissue, which decreases the solubility and permeability of the enamel (13 , 14). As discussed above, several methods are proposed to prevent carie. In most cases, studies on caries prevention are carried out on healthy enamel. However, it would be interesting to understand the effectiveness of these approaches in preventing the progression of caries lesions, that is, to evaluate the effectiveness of different treatments in controlling initial dental caries lesions. Therefore, the objective of the present study was to evaluate the microhardness of white spot lesions treated with different caries prevention methods. The null hypothesis is that treatments are not able to prevent the demineralization of WSL.

## Material and Methods

Fifty bovine incisors without enamel defects were cleaned using a Robinson brush, pumice paste and water. They were then stored in distilled water at 4°C until use. To prepare the 50 enamel samples, the crowns were separated from the roots at the cementoenamel junction. They were subsequently sectioned under water cooling with a diamond disc mounted on a cutting machine (Minitom, Struers A/S, Copenhagen, DK-2610, Denmark). This resulted in 1 sample per tooth crown (5x5x3 mm). To standardize the enamel surface, the samples were polished with 1200, 2400 and 4000 grit sandpaper for 1 minute and, for adequate surface smoothness, they were further polished with a felt disc and 0.5 and 0.03m grit alumina. An area of 16.0 mm² was then delimited with red nail varnish, where the treatments were carried out (Table 1.)

Table 1

- Surface Microhardness Measurement Initially, samples were evaluated for their surface Knoop microhardness (KH) before any intervention (baseline). Specimens were mounted individually in 1-inch acrylic blocks using sticky wax. Five indentations were made in the central area of the sample with a load 50g for 10s and a distance of 100 m between the markings (15). Other KH analyses were performed in a similar manner to what was described above, after the induction of WSL (pH cycling), after the treatments and after the second cariogenic challenge (2nd pH cycling) White spot lesions were induced on dental enamel using a pH cycling model previously described (13). Each sample was fixed on the bottom of 24-well cell culture plates, so that the delimited area was exposed to the demineralizing and remineralizing solutions. The demineralization solution (pH = 5.0) consisted of 2.0 mmol/l Ca and 2.0 mmol/l phosphate in a buffering solution of 0.075mol/l acetate. The remineralization solution (pH= 7.0) consisted of 1.5 mmol/l Ca, 0.9 mmol/l phosphate, 150 mmol/l of potassium chloride. Each specimen was cycled in 3.0 mL of both solutions, being 6 h in the demineralizing solution and 18 h in the remineralizing solution, at 37°C. This procedure was carried out for 14 days at 37°C. Subsequently, a new pH cycle was carried out after the proposed treatments to analyze the therapeutic effectiveness in controlling dental caries. - Treatments The samples of Group 1 (G1) were treated with 2% neutral sodium fluoride gel (2% NaF; Flugel, DFL, Rio de Janeiro, RJ, Brazil), which was applied to demineralized enamel for 3 minutes and then removed with gauze (16). This procedure was repeated after 7 and 14 days. During this treatment interval, the samples remained in distilled water at 37°C. The samples of group 2 (G2) were treated with fluoride varnish (NaF 5%; Duraphat, Colgate-Palmolive, São Bernardo do Campo-SP). The product (0.50g) was placed on the demineralized area using a microbrush for 60 seconds (17). Excess varnish was removed with gauze and the samples were stored in distilled water for 7 days at 37ºC. This procedure was repeated after 7 and 14 days. In group 3 (G3), brushing was carried out with a CPP-ACP based dentifrice (MI PASTE, GC America, Alsip-IL, United States). Brushing was performed with an electric brush (Oral-B Pro-Saúde Power, Procter and Gamble), which was attached to a standardized fixed support. The brush head has 3 sets of bristles of different shapes and positioned at different angles and heights. During brushing, the soft bristles of the brush came into contact with the surface of the samples for 210 seconds, with a force of 1.96N, at room temperature. Assuming that an individual brushes each tooth 3 times a day, during 5 seconds on each side of the tooth, this brushing protocol simulated a total toothbrushing period of 14 days (18). A solution (slurry) was prepared by mixing the dentifrice (Table 1) and distilled water in a 1:2 weight ratio (200 mL of distilled water and 100 g of dentifrice - ISO Specification #145669-1), respectively. During brushing, 1.0 mL of the slurry was injected laterally to the sample, between the restorative material and the toothbrush, every 30 seconds. At the end, the excess dentifrice was removed under running water and the samples were stored in distilled water at 37ºC. The application of the infiltrant resin (Icon - DMG, Chemisch - Pharmazeutische Fabrik GmbH, Hamburg, Germany) was done on specimens of group 4 (G4) according to the manufacturer's instructions. White spot lesions were cleaned and etched with 15% hydrochloric acid (Icon-Etch) for 2 minutes. The acid was removed with water jets and the enamel surface was dried with air jets. Ethanol (99%, Icon-Dry) was applied on WSL for 30 seconds. Then, the surface was dried with air jets and the Icon-Infiltrant resin was applied for 3 minutes and, then, light cured for 40s. A second application of the infiltrant resin was carried out for a period of 1 minute. The surface was polished with felt discs and the samples were immersed in distilled water at 37°C. The samples of group 5 (G5) were irradiated with an Er,Cr:YSGG laser (= 2.78 m; Waterlase, Biolase Technology Inc., San Clemente, CA, USA), with an energy density of 8.92J/cm2, power of 0.5 W, 20 Hz repetition rate, under water cooling, using a sapphire tip of 600 m diameter in a non-contact mode (13). To standardize the irradiation distance, a device was used that attached the laser handpiece to a pre-established distance from the surface of the sample. Irradiation was carried out for 30s, with the fiber positioned perpendicular to the enamel surface and moved in the horizontal and vertical directions, so that the entire pre-established area of 16mm2 received the irradiation. Finally, samples were placed in distilled water and kept at 37ºC. At the end of the treatments, all samples were subjected to a second cariogenic challenge, in the same way as was done previously. The µKH of the samples was analyzed after the treatments and after thesecond pH cycling. Statistical analyses The data were submitted to the D Agostino normality test. One-way ANOVA and Tukey's test, when necessary, were used to analyze the differences in Knoop microhardness numbers (µKHN) of all groups. The level of statistical significance was set at 5%. All analyses were done using Bioestat 5.3 (Instituto Mamirauá, Tefe-AM, Brazil).

## Results

Table 2 shows the (µKHN) of enamel in the initial situation (baseline) and after the white spot lesion induction (1st Cariogenic Challenge), adding the values of all groups.

Table 2

This table shows that the method used to demineralize the enamel samples was effective. It should be noted that no cavitation was observed in the samples after the acid challenge. However, an opaque white area was created, characteristic of the WSL. The longitudinal analysis of enamel hardness in each group is shown in Table 3.

Table 3

It was observed, for group 1 (fluoride gel), that enamel µKHN after treatment (453±13) was similar to that found in baseline (457±15), suggesting that this method was effective in remineralizing enamel. Knoop hardness number analysis after the second cariogenic challenge indicated that fluoride gel increased the acid resistance of the white spot lesion, as in that situation the values were similar to that of the post-treatment situation, but different from those found after the first cariogenic challenge (289±44 ).The longitudinal analysis of the enamel µKHN treated with fluoride varnish (group 2) showed that the treatment recovered the enamel hardness (472±38). As observed for group 1, enamel hardness after the second cariogenic challenge (420±33) was similar to the value found after treatment, but lower than that observed at baseline (498±37). This indicated that the varnish was effective in increasing the acid resistance of the WSL. Samples from group 3 were brushed with CPP-ACP and, unlike G1 and G2, this dentifrice did not remineralize the white spot lesions, nor was able to increase the acid resistance of the WSL. The infiltrant resin (Icon) was used in samples of group 4. Similar to what was observed for G1, the infiltrant recovered the hardness of the demineralized enamel (480±40), and also prevented the demineralization of the white spot lesions (431±66). The same situation was observed in samples irradiated by the Er,Cr:YSGG laser (group 5), where µKHN after treatment (445±47) was statistically similar to baseline (507±85), as well as after the second cariogenic challenge (443±40). Table 4 shows the percentage of hardness recovery of the enamel samples in all groups.

Table 4

This percentage was calculated by comparing the hardness of the treated enamel with that of the sound enamel (baseline). In general, the recovery of enamel hardness was above 86%, that is, the baseline µKHN was always greater than the treated value. It was observed that gel (98.9±0.9%) and icon (97,9±0.5%) presented slightly better results than the CPP-ACP dentifrice (86,7±2.0%) and Er,Cr:YSGG laser (87.7±0.5%). The percentage of acid resistance of the white spot lesion is also shown in table 4. This percentage was calculated by comparing the hardness of the treated enamel with that of the 2nd cariogenic challenge. The data showed that the µKHN of the enamel after the cariogenic challenge was always lower than after treatments (values less than 100%). In groups 1, 3 and 5 the loss of hardness was lower than that observed in groups 2 and 4.

## Discussion

The methods used in this research for the prevention of white spot lesions were able to fully or partially restore the microhardness of demineralized enamel and, in some cases, increase the acid resistance of the lesions. Therefore, the null hypothesis stating that the treatments would not prevent demineralization was rejected. Fluoride compounds enable a conservative approach to enamel demineralization, preventing the destruction of dental structure or even the need for tooth restoration (19). In the present study, the application of fluoride gel (Group 1 - NaF 2%) and fluoride varnish (Group 2 - NaF 5%) on previously demineralized enamel was evaluated. The results showed that both compounds were able to recover the Knoop microhardness (KHN) of the dental tissue after application. Additionally, after a second cariogenic challenge, white spot lesions previously treated with these fluoride agents showed greater resistance to demineralization (Table 3), in agreement with previous findings in the literature (4). The efficacy of fluoride in enamel remineralization is mainly due to its ability to promote the formation of fluorapatite, a mineral less soluble than hydroxyapatite, as it reduces the enamel's critical pH to 4.5, while sound enamel begins to demineralize when salivary pH reaches 5.5 (20). However, higher fluoride concentrations may promote remineralization of the superficial layer of the carious lesion, restricting the penetration of fluoride ions into the body of the lesion due to mineral precipitation that seals the external porosity (20). This characteristic may explain the results of the present study, as the comparison of treatments showed that fluoride gel performed better than varnish after a new cariogenic challenge (Table 4). CPP-ACP (casein phosphopeptide-amorphous calcium phosphate) is a complex derived from casein, a milk protein that binds to enamel and releases calcium and phosphate as needed (8). The released ions penetrate the enamel crystals, increasing their density and hardness, ensuring supersaturated levels, favoring hydroxyapatite formation and remineralization (8 , 21). However, compared to the other treatments, this group showed inferior performance. This may be justified by the short exposure of the samples to CPP-ACP, considering that the application was carried out through a single brushing, whereas the literature suggests that for effective remineralization, prolonged use of MI Paste for 4 weeks is recommended (22). Additionally, the combination of fluoride with the material (CPP-ACPF - MI Paste Plus) generally shows better results (23), but in the present study, this version was not used, as the objective was to test a fluoride-free compound. The infiltration technique with low-viscosity resin penetrates the porosities of demineralized enamel and restores tissue morphology, preventing acid diffusion and improving dental aesthetics by masking the whitish appearance (10). After resin application, an increase in enamel microhardness is observed, as the material fills lesion porosities and makes the structure more cohesive (24). However, despite this performance, a reduction in acid resistance and, consequently, in microhardness was observed after a new acid challenge, which may be related to the material composition, predominantly containing low-molecular-weight monomers (TEGDMA), known for their high hydrophilicity, a characteristic that reduces material hardness in acidic environments and increases polymerization shrinkage (25). The Er,Cr:YSGG laser is used in minimally invasive caries therapy due to its affinity for water and hydroxyapatite in hard tissues. Previous studies have shown that erbium lasers increase the acid resistance of enamel and dentin by promoting tissue heating/fusion and altering their chemical structure to form crystals more resistant to acidity (26 , 27). To avoid enamel ablation, irradiation was performed with low energy density (8.92J/cm²) and without water cooling, as described in the literature (13). According to Table 4, the Er,Cr:YSGG laser recovered nearly 88% of the initial hardness of the samples, while the resistance to demineralization of the irradiated lesions was 99%. This effect can be explained by the rapid heating and fusion of enamel during irradiation, which alters its morphology, obliterating the pores present in white spot lesions and preventing acid penetration (28). Since enamel pores are filled with air, the elimination of these porosities and their replacement by mineralized tissue may increase Knoop hardness values in the irradiated area (28). The results of this study show that all methods were effective in recovering dental enamel hardness. Fluoride varnish and CPP-ACP showed lower potential for preventing white spot lesion demineralization after a new cariogenic challenge. Overall, the recovery of initial enamel microhardness after treatments was greater than 86%, and the methods effectively prevented lesion demineralization with efficacy above 88%. The effectiveness of these methods can be enhanced when combined, for example, by irradiating surfaces with a laser or applying infiltrant resin followed by brushing with fluoride-containing or CPP-ACP dentifrices. Therefore, further studies are needed to evaluate the synergy between these techniques to achieve greater effectiveness in preventing dental caries.

## Conclusions

Considering the limitations of this in vitro study, it is concluded that all methods tested here were able to recover the hardness of tooth enamel, but toothbrushing with a CPP-ACP dentifrice appear not to have the same effectiveness in protecting WSL in a new cariogenic challenge than the other techniques. However, fluoride gel, E,Cr:YSGG laser and the resinous infiltrant were able to keep the hardness of dental enamel to levels similar to its baseline. Although the tested methods did not fully prevent the demineralization of white spot lesions in tooth enamel, their effectiveness was higher than 85%.

## Figures and Tables

**Table 1:** Experimental groups (ED – Energy Density; P- Power).

Groups (n=10)	Treatments
1	Fluoride Gel (NaF 2%)
2	Fluoride Varnish (NaF 5%)
3	MI Paste (CPP-ACP) Dentifrice
4	Icon Infiltrant Resin
5	Er,Cr:YSGG Laser (ED= 8.92 J/cm2, P= 0.5 W)

1

**Table 2:** Mean Knoop microhardness numbers (µKHN) ± standard deviation of enamel found in all groups, considering the initial analyzes (Baseline) and after artificial white spot lesion induction (1st cariogenic challenge).

	Baseline	1st Cariogenic Challenge	p-value
µKHN	495±90	337±71	< 0.0001

2

**Table 3:** Mean Knoop microhardness numbers (µKHN) ± standard deviation of enamel found in each group. Different letters on the same line represent differences statistically significant ( <0.05).

Groups	Baseline	1st Challenge	Treatment	2nd Challenge	p-value
1	457±15a	289±44b	453±13a	428±79a	0.009
2	498±37a	339±51c	472±38ab	420±33b	< 0.0001
3	516±51a	386±92c	448±10b	439±11b	0.0046
4	490±76a	334±82b	480±40a	431±66a	0.0003
5	507±85a	343±41b	445±47a	443±40a	< 0.0001

3

**Table 4:** Percentage of recovery of initial µKHN (treatment/baseline) and percentage of demineralization resistance of white spot lesions (2nd cariogenic challenge/treatment). Different letters in the same column represent differences statistically significant ( <0.05).

Group	Recovery of initial µKHN (%)	Acid resistance of white spot lesion (%)
1	98.9±0.9a	94.4±0.5a
2	94.7±1.0ab	88.9±1.6b
3	86.7±2.0b	97.9±1.2a
4	97.9±0.5a	89.7±1.6b
5	87.7±0.5b	99.5±0.8a
p-value	0.004	0.03

4
